# A fungal ubiquitin ligase and arrestin binding partner contribute to pathogenesis and survival during cellular stress

**DOI:** 10.1128/mbio.00981-24

**Published:** 2024-09-05

**Authors:** Lukas M. du Plooy, Calla L. Telzrow, Connie B. Nichols, Corinna Probst, Natalia Castro-Lopez, Floyd L. Wormley, J. Andrew Alspaugh

**Affiliations:** 1 Department of Medicine, Duke University School of Medicine, Durham, North Carolina, USA; 2 Department of Molecular Genetics and Microbiology, Duke University School of Medicine, Durham, North Carolina, USA; 3 Department of Biology, University of Texas at San Antonio, San Antonio, Texas, USA; 4 Department of Biology, Texas Christian University, Fort Worth, Texas, USA; 5 Department of Cell Biology, Duke University School of Medicine, Durham, North Carolina, USA; Yonsei University, Seoul, South Korea

**Keywords:** *Cryptococcus neoformans*, arrestin, E3 ubiquitin ligase, fungal pathogenesis

## Abstract

Cellular responses to external stress allow microorganisms to adapt to a vast array of environmental conditions, including infection sites. The molecular mechanisms behind these responses are studied to gain insight into microbial pathogenesis, which could lead to new antimicrobial therapies. Here, we explore a role for arrestin protein-mediated ubiquitination in stress response and pathogenesis in the pathogenic fungus *Cryptococcus neoformans*. In a previous study, we identified four arrestin-like proteins in *C. neoformans* and found that one of these is required for efficient membrane synthesis, likely by directing interaction between fatty acid synthases and the Rsp5 E3 ubiquitin ligase. Here, we further explore *Cn* Rsp5 function and determine that this single Ub ligase is absolutely required for pathogenesis and survival in the presence of cellular stress. Additionally, we show that a second arrestin-like protein, Ali2, similarly facilitates interaction between Rsp5 and some of its protein targets. Of the four postulated *C. neoformans* arrestin-like proteins, Ali2 appears to contribute the most to *C. neoformans* pathogenesis, likely by directing Rsp5 to pathogenesis-related ubiquitination targets. A proteomics-based differential ubiquitination screen revealed that several known cell surface proteins are ubiquitinated by Rsp5 and a subset also requires Ali2 for their ubiquitination. Rsp5-mediated ubiquitination alters the stability and the localization of these proteins. A loss of Rsp5-mediated ubiquitination results in cell wall defects that increase susceptibility to external stresses. These findings support a model in which arrestin-like proteins guide Rsp5 to ubiquitinate specific target proteins, some of which are required for survival during stress.

**IMPORTANCE:**

Microbial proteins involved in human infectious diseases often need to be modified by specific chemical additions to be fully functional. Here, we explore the role of a particular protein modification, ubiquitination, in infections due to the human fungal pathogen *Cryptococcus neoformans*. We identified a complex of proteins responsible for adding ubiquitin groups to fungal proteins, and this complex is required for virulence. These proteins are fungal specific and might be targets for novel anti-infection therapy.

## INTRODUCTION

Microorganisms must effectively employ adaptive cellular responses to survive in diverse and rapidly changing environments. The infected host environment is an especially challenging site requiring microbial pathogens to quickly adapt to host conditions including elevated temperatures, nutrient deprivation, and immune responses. Cellular responses to external changes can be accomplished rapidly by post-translational modification of specific proteins, altering their catalytic activity, localization, or regulated protein stability (1–3).

Ubiquitination is one means of post-translational modification providing a very dynamic and versatile process to regulate protein function (4). Traditionally associated with regulated protein stability, the addition of one or more 76-amino acid ubiquitin (Ub) polypeptides to target proteins can also signal for the internalization and altered localization of cell surface proteins in response to various signals. For example, the *Saccharomyces cerevisiae* (*Sc*) general amino acid permease Gap1 is ubiquitinated by an E3 Ub ligase and adaptor protein complex and subsequently internalized when preferred nitrogen sources are present (5, 6). Gap1 is then trafficked via the multivesicular body sorting pathway and degraded in the vacuole or de-ubiquitinated to be recycled back to the plasma membrane when preferred nitrogen sources become limited again so that amino acids can be scavenged for nitrogen (5–7). In another example, the chromatin remodeler Isw1 in *Cryptococcus neoformans* (*Cn*) is ubiquitinated and degraded when cells are exposed to the antifungal drugs fluconazole and 5-fluorocytosine, leading to the differential expression of drug resistance genes (8). The Isw1 protein is not degraded when this chromatin remodeler is acetylated, demonstrating the widespread effect of post-translational regulation on stress response. Ubiquitination is thus a critical component of stress response in modulating protein abundance and protein activity through protein degradation as well as degradation-independent mechanisms.

Since ubiquitination is often associated with the degradation of proteins, it is a process that requires tight regulation. Unsurprisingly, several proteins interact with a Ub moiety before it is ultimately transferred to a targeted protein by a Ub ligase enzyme, and the members of this ubiquitination cascade are themselves transcriptionally and post-transcriptionally regulated (see reference 9 for a review).

One such Ub ligase, the *Sc* Rsp5 (repressor of spt3 phenotype 5) E3 Ub ligase, belongs to the HECT (homologous to the E6-AP carboxyl terminus) class of Ub ligases (10, 11). It is encoded by a constitutively expressed and essential gene, and it plays a major role in heat stress response in *Sc* (12). For instance, during heat stress, *Sc* Rsp5 mediates the endocytosis of several membrane proteins, such as the hexose transporter Hxt3 and the proton pump Pma1. This protects the cell from proteotoxic stress by preventing the over-accumulation of integral proteins in the plasma membrane (13).

Previous work suggests that the predicted Rsp5 ortholog in *Cryptococcus neoformans*, an opportunistic fungal pathogen of humans, plays a similar role in stress response to mammalian body temperature (14). Although *Cn* Rsp5 is not essential for survival of this pathogen in routine laboratory culture conditions, this protein is required for fungal survival at elevated temperatures and during mammalian infection. These characteristics of *Cn* Rsp5 make *Cn* a valuable model to study Rsp5-mediated ubiquitination and to elucidate its role in stress response and fungal pathogenesis.

*Cryptococcus neoformans* grows in the environment in a yeast form, primarily in decaying vegetation and in bird excreta (15). It is an important cause of infection in immunocompromised individuals, especially those with advanced human immunodeficiency virus (HIV) infection, leading to potentially lethal pneumonia and meningoencephalitis if left untreated (16, 17). More than 150,000 cases of cryptococcal meningitis among immunocompromised patients are reported globally every year, accounting for around 19% of all acquired immunodeficiency syndrome-related deaths and with a fatality rate of around 75% in resource-limited areas (18, 19). A better understanding of the pathogenesis of this fungal pathogen could consequently lead to the development of more accessible treatment options in low-resource regions plagued by poorly treated HIV infection.

Ubiquitin ligases, including *Sc* Rsp5, often interact with target proteins indirectly and rely on the binding of an adaptor protein to recognize their targets (20). This interaction with adaptor proteins serves to regulate the activity of these Ub ligases. Characterization of several adaptors for Rsp5 in *Sc* revealed that these “bridges” between Rsp5 and its target are catalytically inactive and often contain arrestin domains, homologous to the arrestin domains first identified in the mammalian visual β-arrestins that “arrest” the rhodopsin receptor protein and in turn mediate sight in animals (21, 22). The related α-arrestins that bind Rsp5 and its homologs are ubiquitous in most eukaryotes except for plants, but their primary amino acid sequences typically diverge between species. This divergence limits their identification through homology-directed screens (23, 24). Additionally, other non-arrestin proteins also serve as adaptor proteins for Rsp5 interactions, which, along with the fact that Ub ligase interactions are very transient, complicates the identification of targets of Rsp5 with conventional interaction studies (25–27).

In a previous study, we identified four *Cn* α-arrestin-like proteins (Ali1, Ali2, Ali3, and Ali4) that mediate stress response phenotypes (28). We also showed that these four *Cn* Ali proteins contain PxY motifs, which associate with the tryptophan-tryptophan (WW) domains of E3 Ub ligases (29). Additionally, Ali1 and Ali2 contain two arrestin domains, both an N- and a C-terminal arrestin domain, akin to the human α-arrestin proteins. One of the *Cn* arrestin-like proteins, Ali1, contributes to efficient cell division during stress by recruiting fatty acid synthases (Fas1 and Fas2) to the sites of cell division, therefore mediating the abundance of these synthases to regions requiring rapid membrane synthesis (28). The specific roles of protein targets bound by the other α-arrestin-like proteins during *Cn* stress response remain to be determined. In the current study, we further examine the role of *Cn* α-arrestins and their Ub ligase binding partner, Rsp5. By demonstrating common interacting proteins, we confirm a model of interaction between the *Cn* Rsp5 E3 Ub ligase and the α-arrestin-like adaptor protein *Cn* Ali2. We also explore the roles of Rsp5 and arrestin-mediated ubiquitination on target protein stability and function, as well as the physiological consequences for cell stress responses and pathogenesis.

## RESULTS

### The *Cn* Ali2 arrestin-like protein interacts with the Rsp5 E3 Ub ligase

**Fig 1:**
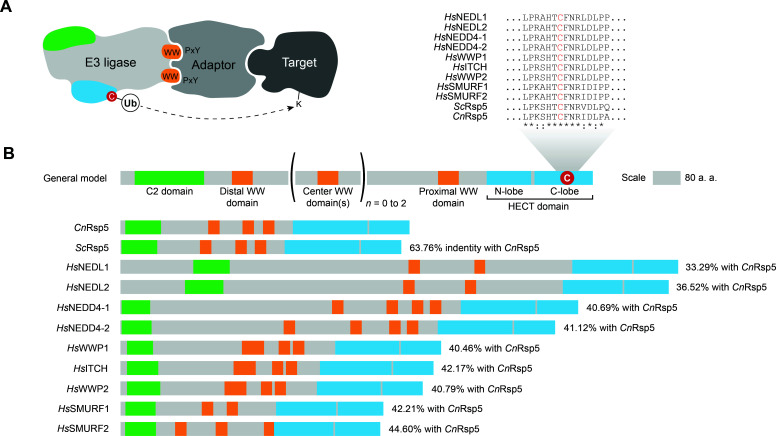
(A) Proposed model of the interaction of a Nedd4-like E3 ubiquitin ligase (such as *Cn* Rsp5) and its target protein facilitated by an adaptor protein (such as an arrestin protein). The interaction is mediated through WW domains in the E3 ligase and PxY motifs in the adaptor protein. (B) Despite significant overall protein sequence divergence, cysteine residues in the E3 ligase HECT domain required for ubiquitin transfer are conserved in *Cn* Rsp5, *Sc* Rsp5, and the Nedd4 family of E3 ligases in humans. Other conserved functional domains are graphically indicated.

In our earlier study, we identified the putative Rsp5 Ub ligase (encoded by CNAG_05355) as one of the strongest interactors with the *Cn* Ali1 arrestin-like protein (28). We hypothesized that Ali2 would also interact with the putative *Cn* Rsp5, as both of these arrestin-like proteins share homology with Rsp5-binding arrestin proteins in *Sc*. The *Cn* Ali1 and Ali2 proteins share 27% protein sequence identity while no significant protein sequence homology is found between Ali1 and either Ali3 or Ali4 (30, 31). Finally, both *Cn* Ali1 and Ali2 proteins contain at least two C-terminal PxY motifs, which are known to bind to WW domains in E3 Ub ligases, suggesting that both of these arrestin-like proteins serve as adaptors for E3 Ub ligases (Fig. 1A) (32).

To test this hypothesis, we used a proteomics-based mass spectrometry approach to identify potential protein interactors of Ali2. To do so, we performed green fluorescent protein (GFP) immunoprecipitation of total cell lysates of the wild-type (WT) strain (as a negative control) and an *ali2*Δ mutant strain complemented with the GFP-tagged *ALI2* gene (*ali2*Δ + *ALI2-GFP*). A total of 651 proteins were identified as potential Ali2-GFP interactors using this approach. We prioritized proteins that were highly represented in the *ali2*Δ + *ALI2-GFP* immunoprecipitations and minimally represented, if at all, in the WT immunoprecipitation. Using this methodology, we identified 41 statistically relevant potential interactors of Ali2-GFP (Table S1) with the highest average exclusive unique peptide counts (APC) in the *ali2*Δ + *ALI2-GFP* immunoprecipitations (used as a measure of interaction strength) and the lowest APC percent in the WT immunoprecipitation (used as a measure of interaction specificity). Of all the identified potential interactors, Rsp5 had the highest APC in the *ali2*Δ + *ALI2-GFP* immunoprecipitations (~29) and a relatively low APC percent in the WT immunoprecipitation (~3%). These observations suggest that Rsp5 is a strong interactor with Ali2.

To gain insight into the cellular processes in which Ali2 might be involved, we used the computed gene ontology (GO) terms provided on the Eukaryotic Pathogen, Vector, and Host Informatics Resource (VEuPathDB) Fungal database (FungiDB) to infer the molecular function of the remaining interactors (33). Twenty-six of the remaining 40 statistically significant potential interactors of Ali2-GFP are ribosomal or nucleic acid-binding proteins. Although this interaction with ribosomal proteins or nucleic acids may be biologically relevant, ribosomal and nuclear proteins are often enriched during interactome screens as they interact with the bait protein during translation (34).

In *Sc*, the Rsp5 Ub ligase is encoded by an essential gene, while previous work has suggested that *Cn* Rsp5 is non-essential for growth in routine laboratory conditions (14). Similarly, disruption of individual members of the mammalian Nedd4 family of Ub ligases, the closest mammalian homologs to *Sc* Rsp5, results in several physiological defects in mice, such as severe immunological defects (in the case of the Itch ligase) and embryonic lethal heart defects (Nedd4-1 ligase) (35, 36). We aligned the putative *Cn* Rsp5 E3 Ub ligase protein sequence with the *Sc* Rsp5 and the human Nedd4 family of Ub ligases sequences using the European Bioinformatics Institute (EMBL-EBI) Clustal 2.1 protein identity matrix alignment algorithm to determine the conserved status of this predicted Rsp5 ligase. We identified a calcium-dependent phospholipid-binding C2 domain, three PxY motif-binding WW domains, and a C-terminal HECT domain, similar to Rsp5 in *Sc* (37, 38). The Nedd4/Rsp5 family of Ub ligases have a similar domain organization but differ in sequence homology and length, with the only highly conserved region among the fungal Rsp5 ligases and the human Nedd4 family occurring along a short stretch of residues flanking the catalytic cysteine in the HECT domain (Fig. 1B). Therefore, despite similar predicted underlying enzymatic functions between the fungal and mammalian homologs of Rsp5, there are likely fungal-specific differences in regulation and protein targets of these Ub ligases. This is further supported by a previous study that showed expression of a human Nedd4-1 protein in *Sc* could not restore viability due to deletion of *RSP5* in *Sc* (39).

### *Cn* Ali2 and Rsp5 are important for survival during stress

**Fig 2:**
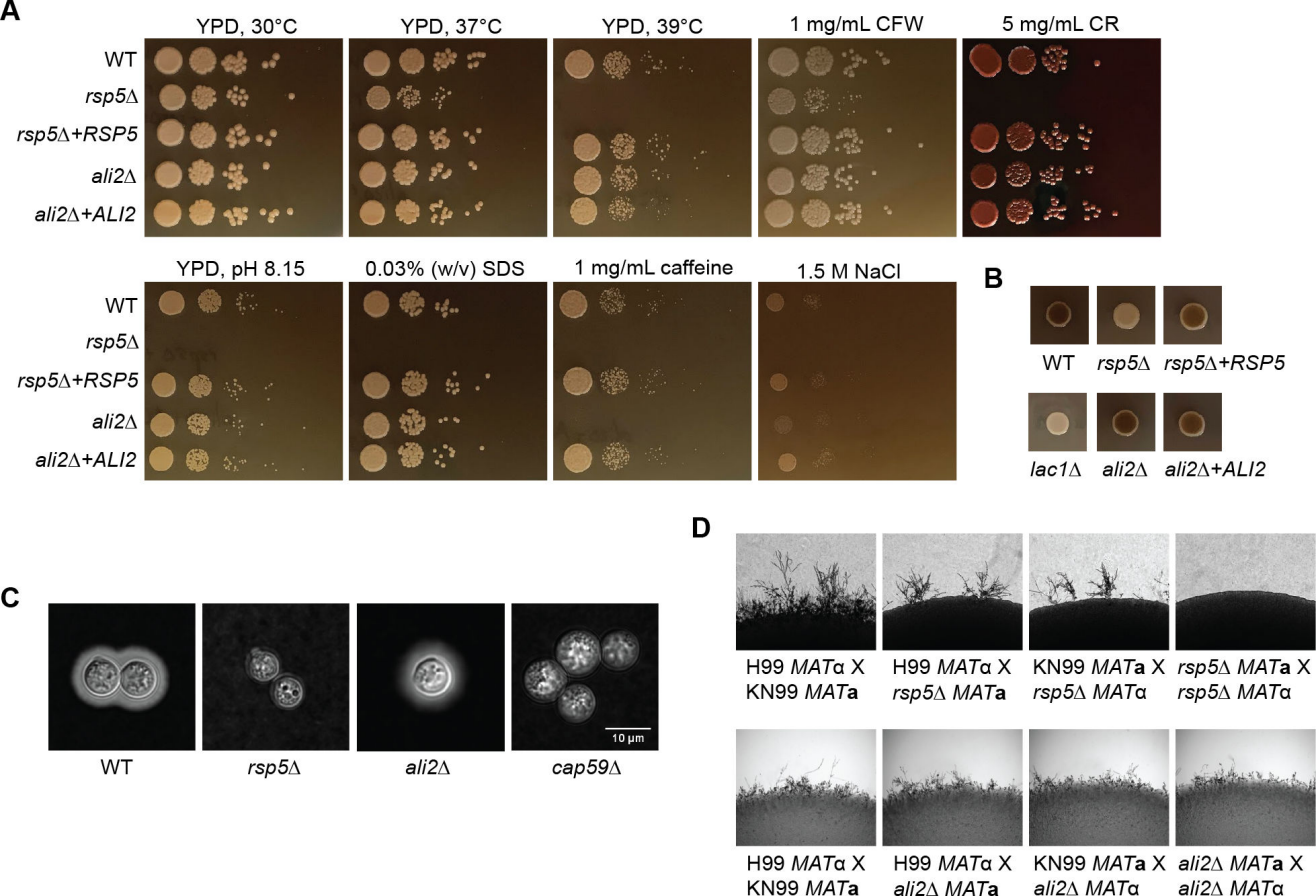
*Cn* Rsp5 and Ali2 proteins are required for overlapping stress response phenotypes and cellular differentiation. (A) Indicated strains were incubated in serial spot dilutions on rich media (yeast extract-peptone-dextrose [YPD]) at different temperatures, as well as YPD medium containing various cell wall stressors. Colony growth was assessed at 2 to 4 days. (B) Melanin activity indicated by brown colony color was assessed on L-Dopa medium at 2 days of incubation at 30°C for each indicated strain. (C) The WT, *rsp5*Δ, and *ali2*Δ mutants were incubated in capsule-inducing conditions (CIM) for 4 days at 37°C. Surface capsule was assessed by India ink counter-staining (600×) with the acapsular *cap59*Δ mutant as a control. (D) The indicated MATα and MATa strains were co-incubated in mating patched on MS mating medium for 7 days. Filamentation at the edge of the mating was assessed as a marker for cellular differentiation associated with mating.

Since our protein-protein interaction study suggested that *Cn* Ali2 interacts with Rsp5, we hypothesized that Ali2 and Rsp5 may both be required for overlapping processes of adaptation to specific stressors that are relevant to the pathogenesis of this fungus. To explore potential functional relationships between cryptococcal Ali2 and Rsp5, we made use of loss-of-function *rsp5*Δ and *ali2*Δ mutant strains and compared the growth phenotypes of these strains in the presence of various cell surface stressors. The *rsp5*Δ mutant strain displayed a marked attenuation of growth in all of these tested stress conditions. In contrast, the *ali2*Δ mutant strain demonstrated growth defects in a subset of these cell stresses, with reduced growth in the presence of 1.5 M sodium chloride (NaCl) and 1 mg/mL caffeine (Fig. 2A). All of the growth defects were restored when the mutant strains were complemented with the respective *Cn RSP5* or *ALI2* genes in the endogenous gene locus.

In addition to the inability to grow in host-relevant temperature, the *rsp5*Δ mutant strain shows alterations in other “classical” pathogenesis factors associated with cryptococcal pathogenesis in mammals. These include a reduced ability to form capsule and melanin, while the *ali2*Δ mutant strain showed WT-like melanin and capsule formation (Fig. 2B and C). Additionally, a unilateral mating cross of the *rsp5*Δ strain with WT resulted in a slight disruption in filamentation and severely disrupted filamentation in an *rsp5*Δ bilateral cross, while no such differences were seen between WT and *ali2*Δ mutant cells (Fig. 2D). These results demonstrate that *Cn* Rsp5 is required for many microbial phenotypes required for pathogenesis and cellular differentiation, and that *Cn* Ali2 mediates a subset of the Rsp5 stress-dependent phenotypes.

### The Pkc1-mediated cell wall integrity pathway depends on Rsp5-mediated ubiquitination

**Fig 3:**
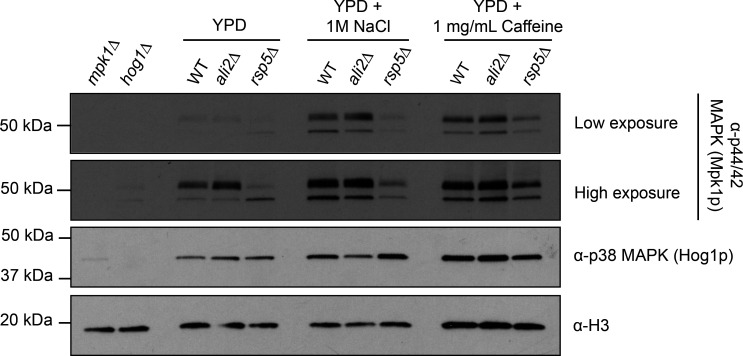
*Cn* Rsp5 is required for Mpk1 stress response pathway activation. The indicated strains were incubated in either yeast extract-peptone-dextrose (YPD) + 1 mg/mL caffeine or YPD + 1 M NaCl medium. Hog1 phosphorylation in total protein lysates was assessed by western blot using an α-phospho-Hog1 monoclonal primary (α-p38 Hog1) antibody with an α-H3 polyclonal primary antibody used for loading control and the *hog1*Δ mutant as negative control. Phosphorylation of Mpk1 was measured by western blotting of total protein lysates from the indicated strains using an α-p44/42 monoclonal primary antibody, an α-H3 antibody as loading control, and the *mpk1*Δ mutant as negative control.

Since both Ali2 and Rsp5 are required for growth in the presence of caffeine, we assessed their roles in the activation of the Pkc1-mediated cell wall integrity stress response pathway. Caffeine is a known inducer of the Pkc1-mediated cell wall integrity pathway and a defective pathway leads to impaired growth in the presence of caffeine (40, 41). We incubated the WT, the *ali2*Δ mutant, and the *rsp5*Δ mutant strains, as well as a *mpk1*Δ mutant strain lacking the mitogen-activated protein kinase 1 (MAPK1) that forms part of the pathway in the presence and absence of 1 mg/mL caffeine as well as 1 M NaCl. We then assessed phosphorylation levels of the Mpk1 protein as a marker of pathway activation via western blotting (42). Although no difference in phosphorylation levels of Mpk1 was observed between WT and the *ali2*Δ mutant, there was marked reduction in Mpk1 phosphorylation seen in the *rsp5*Δ mutant in all tested conditions (Fig. 3).

Similarly, to determine if the growth defects in the presence of high NaCl concentrations seen in the *ali2*Δ and *rsp5*Δ can be attributed to defective activation of the high osmolarity glycerol response pathway, we incubated the WT, the *ali2*Δ mutant, and the *rsp5*Δ mutant strains in the presence and absence of 1 M NaCl and assessed phosphorylation levels of the MAPK Hog1 as a marker of HOG pathway activation (42). In contrast to the major decrease in phosphorylated Mpk1 in the *rsp5*Δ mutant strain, we observed intact induction of Hog1 phosphorylation under both conditions in the WT and both mutant strains (Fig. 3). This finding indicates that Ali2 and Rsp5 are not required for HOG pathway activation in response to NaCl stress, and as a result, the growth defects of the *ali2*Δ and *rsp5*Δ mutant strains in this condition are not due to aberrant HOG pathway signaling. However, impaired Mpk1/Pkc1 may underlie many of the growth phenotypes of the *rsp5*Δ mutant.

### Rsp5-mediated ubiquitination affects cell wall composition and organization

**Fig 4:**
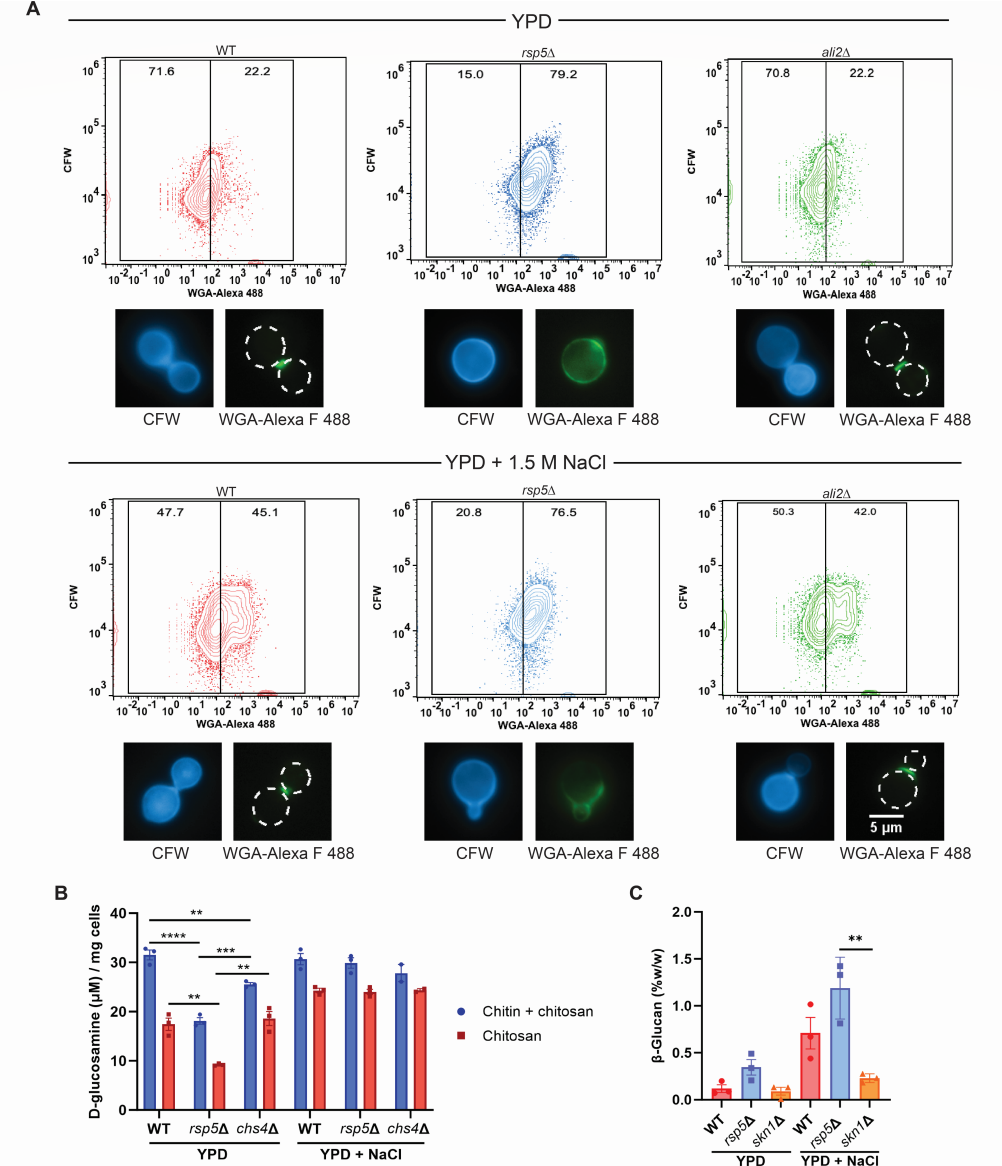
The *rsp5*Δ mutant cells has more exposed chitin and produced less chitin and chitosan overall. (A) WT, *rsp5*Δ, and *ali2*Δ cells were incubated in YPD or YDP with 1.5 M NaCl for 1 h, fixed with formaldehyde, and double-stained with CFW and WGA-Alexa Fluor 488. Stained cells were analyzed with a BD Biosciences FACSCanto Clinical Flow Cytometry System and results were analyzed and plotted with BD Biosciences FlowJo software. Representative fluorescent microscopy images of the double-stained cells are shown for each strain and condition. (B) WT, *rsp5*Δ, and *chs4*Δ (as a positive comparison) cells were incubated in YPD or YPD with 0.5 M NaCl for 48 h. Total chitin and chitosan amounts produced by these cells were assessed with MBTH (3-methyl-benzothiazolinone hydrazine hydrochloride)-based chitin/chitosan quantification (μM glucosamine/10^7^ cells) using lyophilized cell material. Statistical differences were calculated using an unpaired *t*-test. The error bars represent the standard error of the mean and statistical differences are only indicated where they occur (**P < 0.001; ***P < 0.0001; ****P < 0.00001). (C) WT, *rsp5*Δ, and *skn1*Δ (as a positive comparison) were incubated in YPD or YPD with 0.5 M NaCl for 48 h. The total amount of β-glucan produced by these cells was assessed with the Megazyme yeast β-glucan quantification kit using lyophilized cell material. Statistical differences were calculated using an unpaired *t*-test. The error bars represent the standard error of the mean and statistical differences are only indicated where they occur (**P < 0.001).

To further explore whether specific cell wall defects are present in the *rsp5*Δ mutant and associated with its reduced ability to grow in the presence of Congo red (CR) and calcofluor white (CFW), we assessed cell wall β-1,3 and β-1,4 polysaccharide content and degree of chitin/chitosan exposure on the cell wall surface in the *rsp5*Δ and *ali2*Δ mutants compared to WT. The WT, *rsp5*Δ mutant, and *ali2*Δ mutant cells were cultivated in yeast extract-peptone-dextrose (YPD) medium in the absence or presence of 1.5 M NaCl. CFW staining was similar among the three strains in each condition, suggesting similar cell wall β-1,3- and β-1,4-linked polysaccharide content, including chitin, chitosan, and many glucans. As a small molecule, CFW readily infiltrates the cell wall, binding to β-1,3- and β-1,4-linked polysaccharides (43). In contrast, we noted enhanced staining intensity in the *rsp5*Δ mutant strain compared to the WT and *ali2*Δ mutant strain using Alexa Fluor 488 conjugated to wheat germ agglutinin (WGA). This large chito-oligomer-binding lectin is unable to deeply penetrate the fungal cell wall and is therefore a useful marker of changes in chitin/chitosan surface exposure. Importantly, previous studies have determined that increased staining intensity by Alexa Fluor 488-WGA in *Cn* correlates with altered cell wall architecture and increased activation of macrophages as measured by tumor necrosis factor-α production (44). While *ali2*Δ mutant cells grown in either condition displayed no changes in WGA staining as a marker of chitin/chitosan exposure compared to WT, we did observe more exposed chitin/chitosan (measured by Alexa Fluor 488-WGA) in *rsp5*Δ cells in both conditions (Fig. 4A).

We further assessed whether the altered chitin exposure in the *rsp5*Δ mutant was associated with alterations in cell wall carbohydrate content especially two cell wall polysaccharides that might serve a chitin-masking role. In contrast to the *skn1*Δ mutant with a known defect in β-glucan synthesis, biochemical quantification of total cell wall β-glucans demonstrated no statistically significant differences between the WT and *rsp5*Δ mutant incubated either in the presence or absence of 0.5 M NaCl for 48 h (Fig. 4C). In contrast, the *rsp5*Δ mutant had a ~50% reduction in total chitin/chitosan levels compared to WT when incubated in YPD medium, similar to the *chs4*Δ chitin synthase mutant (Fig. 4B) (45). However, in the presence of 0.5 M NaCl, no differences in chitin and chitosan were observed between the WT, *rsp5*Δ, and *chs4*Δ mutant cells. These results suggest that Rsp5 does affect both cell wall composition and organization, perhaps through its regulatory ubiquitinating activity of cell wall-active proteins. However, this indirect effect on cell wall chito-oligomer content is not appreciated in the presence of stresses such as hypertonic salt, perhaps given the considerable cell wall adaptations that occur in response to significant stress conditions (46–48).

### The roles of Rsp5 and Ali2 in pathogenesis

**Fig 5:**
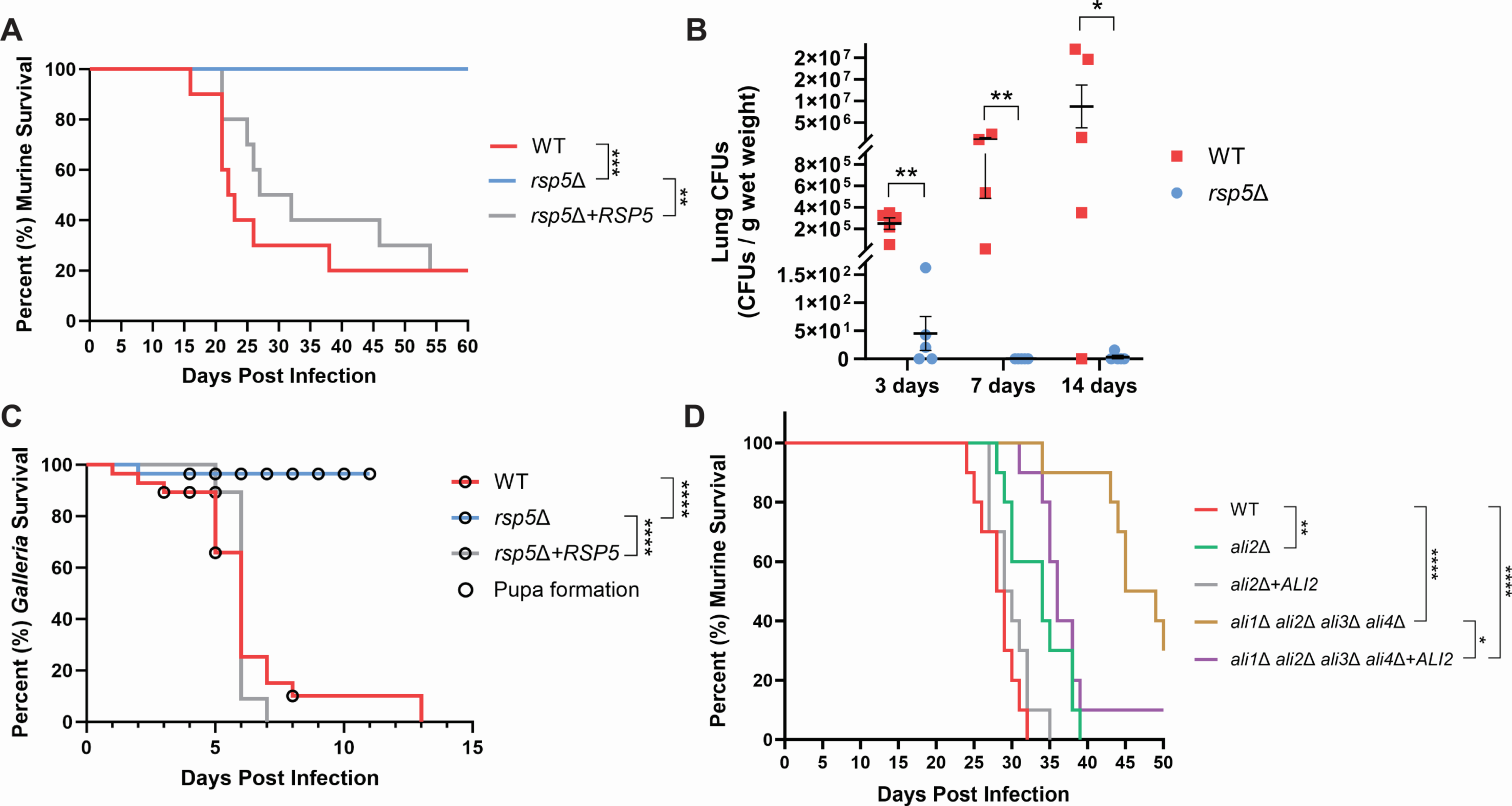
(A) C57BL/6 mice were inoculated by inhalation with either the WT, *rsp5*Δ, or *rsp5*Δ + *RSP5* strains (10 mice per strain) and monitored for clinical signs predicting imminent mortality. Statistical differences between survival curves were calculated using the log-rank (Mantel-Cox) test (**P < 0.001; ***P < 0.0001). (B) C57BL/6 mice were inoculated by inhalation with either the WT or *rsp5*Δ strains. Lung fungal burden at indicated time points was determined by quantitative culture of homogenized harvested tissue (colony-forming units [CFUs] per gram tissue). Each data point represents lung CFUs from individual animals (error bars = standard error of the mean; statistical differences calculated using the Mann-Whitney U-test (*P < 0.01; **P < 0.001). (C) Greater wax moth larvae (*Galleria mellonella*) were inoculated with 10^7^ cells of the indicated *Cn* strains and incubated at 30°C. Mortality was monitored for 20 larvae for each group over 14 days. Statistical differences were calculated using the log-rank (Mantel-Cox) test (****P < 0.00001). (D) BALB/c mice were inoculated by inhalation with indicated *Cn* strains (10 mice per strain) and monitored for clinical signs predicting imminent mortality. Statistical differences between survival curves were calculated using the log-rank (Mantel-Cox) test (*P < 0.01; **P < 0.001; ****P < 0.00001).

Given the alterations in stress-dependent phenotypes of the *ali2*Δ and *rsp5*Δ mutants and the cell wall defects of the *rsp5*Δ mutant, we assessed their relative virulence compared to WT in a C57BL/6 murine inhalation model of cryptococcal infection. This mode of infection in this mouse background typically results in a lethal infection, with dissemination from the lungs to the brain. Mice infected with the WT strain survived a median of 22 days after infection. Similar rates of survival were observed in mice infected with the *rsp5*Δ + *RSP5* complemented strain, with two mice in each group surviving to the end of the experiment, possibly due to incomplete inhalation of the cryptococcal cells. In contrast, infections with the *rsp5*Δ mutant strains resulted in no lethal events during the course of the 60-day period of observation (Fig. 5A).

We subsequently assessed quantitative cultures of the lungs and brain tissue at different time points for inhalation of the WT and the *rsp5*Δ cryptococcal cells. Consistent with prior experiments, we observed a progressive increase in the lung fungal burden of mice infected with the WT strain. At 3 days after infection, there was an average of 2.5 × 10^5^ colony-forming units (CFUs) per gram of lung tissue in five infected mice. This increased to an average of 9.5 × 10^5^ CFUs/g of lung tissue at 7 days, and 8.7 × 10^6^ CFUs/g of lung tissue at day 14. In the *rsp5*Δ mutant, we only recovered 45 CFUs/g lung tissue at day 3, no colonies at day 7, and a single colony from one mouse at day 14 (Fig. 5B). Therefore, not only is the *Cn* Rsp5 protein required for virulence in our murine model, but it is also required for the colonization and survival of *Cn* in mice over long periods of time.

To determine if the inability to grow at elevated temperatures solely drives the avirulence phenotype of the *Cn rsp5*Δ strain, we also infected larvae of the greater wax moth (*Galleria mellonella*) with 10^7^ cryptococcal cells and incubated the larvae at 30°C while following the course of the infection. The WT and *rsp5*Δ + *RSP5* strains showed a median survival of 7 days, while only a single larva infected with the *rsp5*Δ strain died during the 14-day course of the experiment (Fig. 5C). This suggests that virulence-related phenotypes other than the inability to survive at high temperatures are also regulated by *Cn* Rsp5.

In a separate experiment, we inoculated BALB/c mice by inhalation with the WT strain, the *ali2*Δ mutant strain, and the *ali2*Δ + *ALI2-GFP* complemented strain, subsequently tracking murine survival. This mouse strain is relatively resistant to *C. neoformans* infections due to a helper T2 cells-shifted cytokine profile and can be used to distinguish subtle alterations in virulence between fungal strains (49). In these experimental infections, we observed that the *ali2*Δ mutant strain is attenuated in virulence compared to the WT strain, and this defect is rescued by complementation with the *ALI2* gene (Fig. 5D). Mice inoculated with the WT strain and the *ali2*Δ + *ALI2-GFP* strain had a median survival time of 28.5 and 29.5 days, respectively, while those inoculated with the *ali2*Δ mutant strain had a median survival time of 34 days (Fig. 5D). We also assessed the contributions of Ali2 to the virulence defects previously observed in the arrestin null (*ali1*Δ *ali2*Δ *ali3*Δ *ali4*Δ) mutant strain. To do so, in the same experiment, we also inoculated BALB/c mice by inhalation with 10^4^ cells of the arrestin null mutant strain and the arrestin null mutant strain complemented with the *ALI2* gene (*ali1*Δ *ali2*Δ *ali3*Δ *ali4*Δ + *ALI2-GFP*). The arrestin null mutant strain displayed significant virulence attenuation, with 30% of inoculated mice surviving to the end of the experiment. However, only 10% of mice inoculated with the arrestin null mutant strain complemented with the *ALI2* gene survived to the end of the experiment. These results indicate that the addition of Ali2 alone into the arrestin null background results in an increase in fungal virulence, characterized by a more rapid onset of cryptococcal disease and overall increased murine mortality. This observation suggests some degree of functional overlap among the Ali proteins during the infection process, with Ali2 having a more pronounced role for establishing virulence than the other arrestin proteins. Moreover, the apparently more severe virulence attenuation of the *rsp5*Δ mutant compared to individual arrestin mutant strains further supports a model in which individual adaptor proteins mediate a subset of interactions between Rsp5 and its protein targets.

### Ali2 is required for the ubiquitination of a subset of the targets of Rsp5

**Fig 6:**
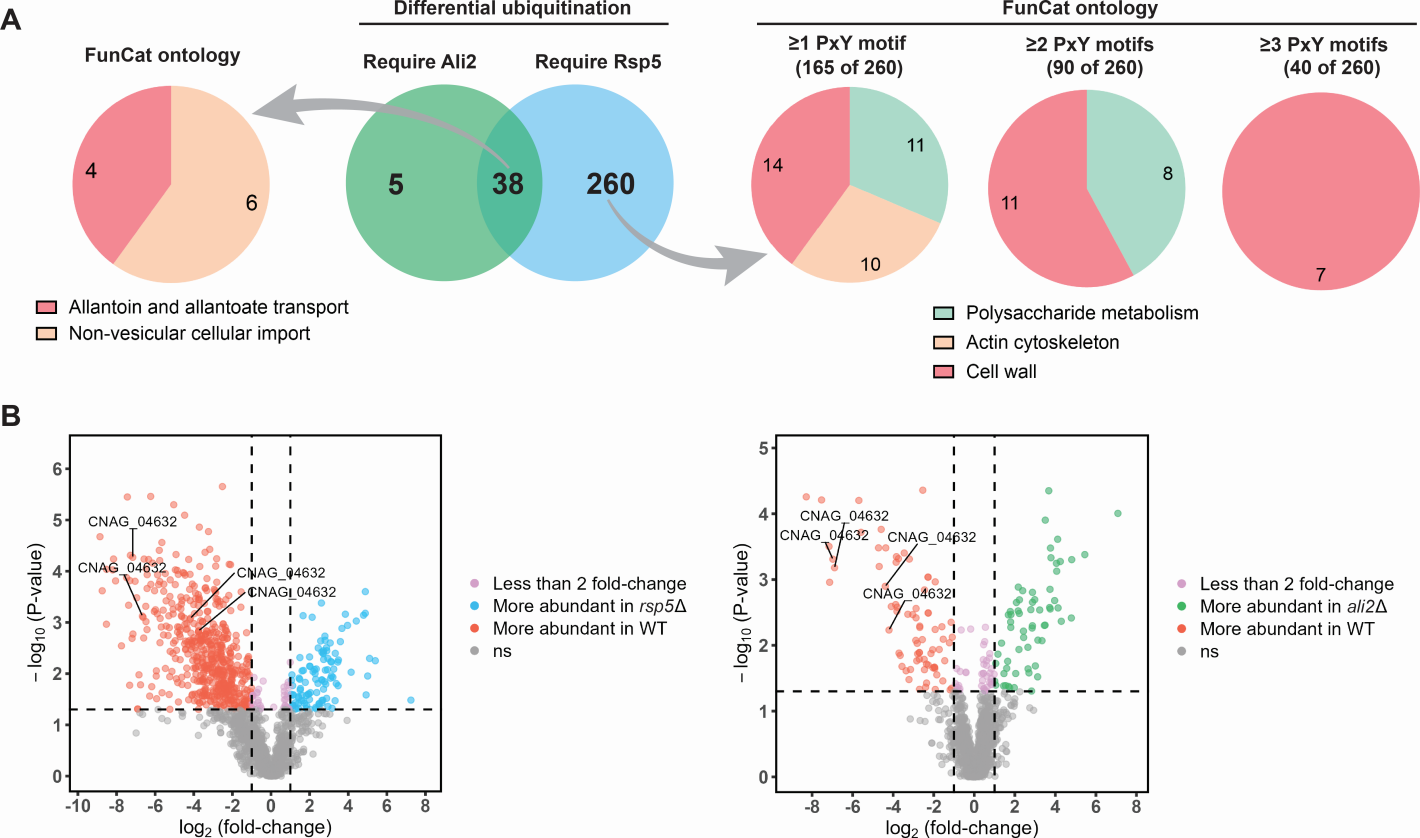
Identification of proteins requiring Rsp5 or Ali2 for ubiquitination. The WT, *rsp5*Δ, and *ali2*Δ strains were incubated in either YPD medium or YPD + 1.5 M NaCl for 1 h. Total cell lysates were treated with trypsin, creating a ubiquitin remnant stump in ubiquitinated proteins. These previously ubiquitinated proteins were enriched by immunoaffinity using the PTMScan HS Ubiquitin/SUMO Remnant Motif (K-ε-GG) kit and identified by quantitative liquid chromatography-tandem mass spectrometry (LC/MS/MS). Proteins ubiquitinated in the WT strain and not in either the *rsp5*Δ or *ali2*Δ mutant strain were quantified. (A) Venn diagram indicating the number of proteins requiring either Rsp5 (298), Ali2 (43), or both (38), for ubiquitination (middle panel). Predicted protein function for proteins requiring both Rsp5 and Ali2 (left panel), or those only requiring Rsp5 (right panel), was assessed using the FunCat ontology algorithm. Protein ontology is indicated for protein subdivided by number of PxY motifs present in the protein sequence (right panel). (B) Volcano plot of individual peptides identified by mass spectroscopy demonstrating relative peptide abundance (log_2_ fold change) in the *rsp5*Δ (left panel) or *ali2*Δ (right panel) strains compared to WT. The protein encoded by the gene CNAG_04632 is represented by several unique peptides (indicated data points).

We used a proteomics-based differential ubiquitination screen to identify targets of Rsp5 and Ali2 that might explain the growth impairments of the *rsp5*Δ and *ali2*Δ strains during stress. We exposed cells to 1.5 M NaCl for 1 h, a condition in which both the *rsp5*Δ and *ali2*Δ mutants were growth impaired. After trypsin digestion, ubiquitinated proteins are marked by a unique ubiquitin diglycine remnant stump (K-ε-GG) that was recognized by a specific antibody-mediated enrichment strategy followed by identification and quantification by mass spectroscopy (50). From this screen, we identified 298 unique proteins that are more abundant in a ubiquitinated form in WT compared to *rsp5*Δ (Fig. 6A; Table S2). Likewise, 43 proteins were more abundant in the ubiquitinated form in WT lysates compared to the *ali2*Δ cell lysates (Fig. 6A; Table S3). When comparing these sets, we found 38 overlapping proteins that were more abundant in the WT cell lysates compared to both the *rsp5*Δ and *ali2*Δ cell lysates, indicating a significant common core group of proteins that require both Rsp5 and Ali2 for ubiquitination. The *Cn* Pkc1 protein, a protein kinase induced during cell wall stress and a signaling partner of the Mpk1 kinase, also requires Rsp5 for its ubiquitination, as revealed by this screen. In conjunction with our finding that Mpk1 is less abundant in a phosphorylated form in the *rsp5*Δ mutant than in WT and *ali2*Δ mutant cells, these results suggest that Rsp5 might affect phosphorylation of Mpk1 through ubiquitination of Pkc1.

To predict functions enriched among the proteins that require both Rsp5 and Ali2 for ubiquitination, we used the FungiFun2 systematic ontology analysis tool specifically developed for fungi (51). This analysis predicted allantoin and allantoate transport as the most highly represented term and is represented by four genes, including CNAG_04632, encoding the most differentially ubiquitinated protein that requires both Ali2 and Rsp5 for its ubiquitination. A volcano plot (Fig. 6B) demonstrates the degree of enrichment of peptides aligning to this protein relative to other peptides that require either Rsp5 or Ali2 for their ubiquitination.

There are 260 proteins that require Rsp5 and not Ali2 for ubiquitination. This likely explains the more severe mutant growth phenotypes of the rsp5Δ mutant compared to the ali2Δ mutant. Of the 260 proteins that require Rsp5 ubiquitination, the majority (165 of 260) contain at least one PxY motif known to facilitate interactions between E3 Ub ligases and their targets, 90 have at least two PxY motifs, and 40 have at least three of these motifs (Fig. 6A). This observation suggests that *Cn* Rsp5 protein ubiquitination is likely facilitated by both arrestin-dependent and arrestin-independent means. The functional categories represented by Rsp5 targets with multiple PxY motifs are highly enriched for cell wall modifying enzymes (Fig. 6A), including those involved in the synthesis of cell surface components, such as α- and β-glucan (Skn1 and Fks1, respectively) and chitin (Chs4 and Chs5). Together, these observations may explain the increased susceptibility of the *rsp5*Δ mutant to cell wall perturbations compared to the *ali2*Δ strain (Fig. 2A, 4A and B). Several nutrient importers, such as the proton-dependent oligopeptide transporter (Ptr2), known to be ubiquitinated by Rsp5 in *Sc*, were also identified in our screen (52).

All of the peptides mapping to the Ub peptide in our screen have a diglycine residue on the 63rd lysine residue, indicating that *Cn* Rsp5 catalyzes the formation of K63-linked polyubiquitin chains, as is also known about *Sc* Rsp5 (53). In *Sc*, this polyubiquitin-chain topology directs ubiquitinated proteins into the multivesicular pathway and ultimately into the vacuolar lumen for degradation or resorting (54). Our screen further indicates that, like *Sc* Rsp5, *Cn* Rsp5 also ubiquitinates and, therefore, interacts with the Ubp5 deubiquitinase, an ortholog of Ubp15 in *Sc*, that serves a regulatory role through the antagonization of Rsp5 activity (55).

### Ubiquitination and degradation of specific Rsp5 targets is mediated by *Cn* α-arrestin-like adaptors

**Fig 7:**
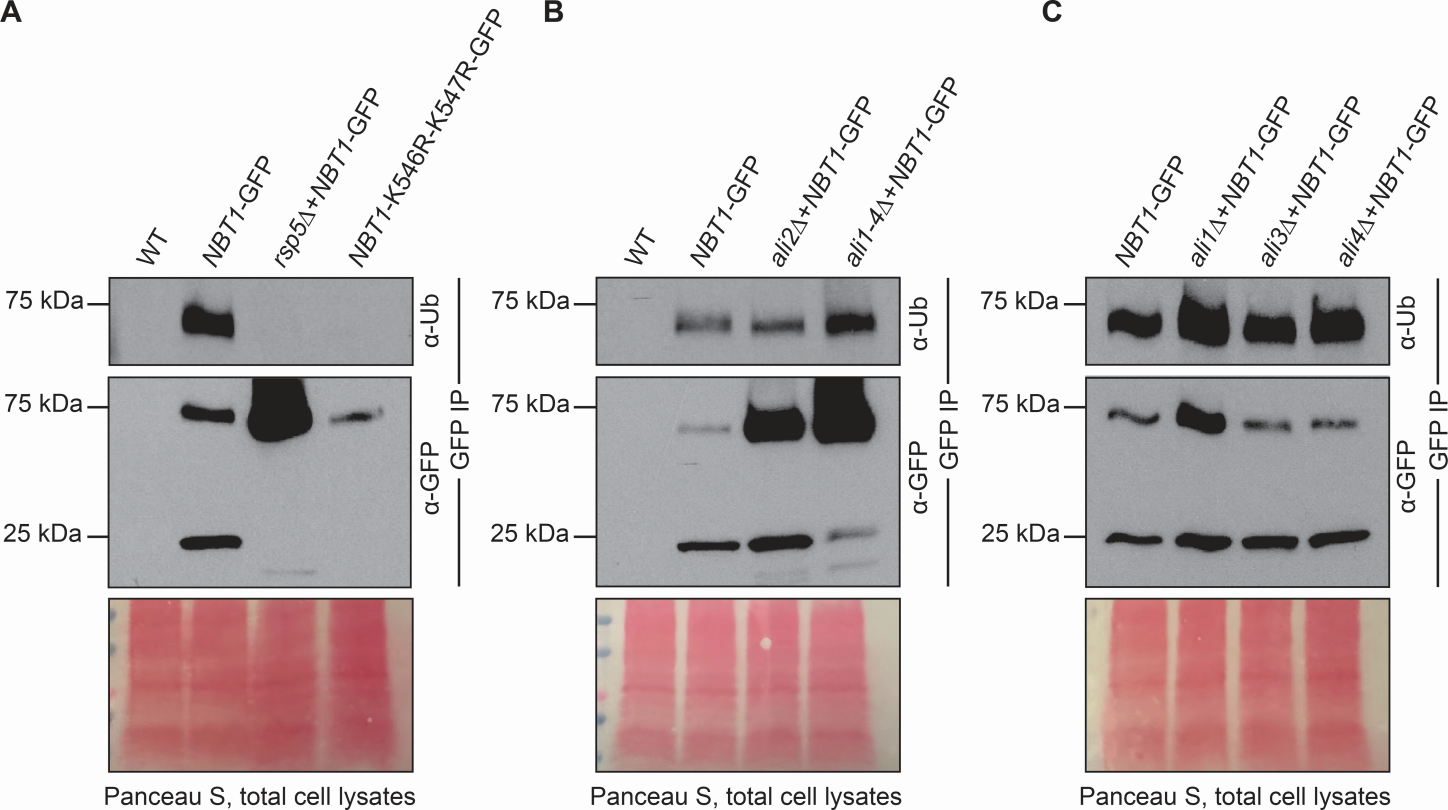
The indicated strains were incubated in YPD medium overnight (16 h). Total cell lysates were enriched for GFP-tagged proteins by immunoaffinity using GFP-trap (ChromoTek) resin. Eluted proteins from this immunoprecipitation were assessed by western blot using either an an α-GFP monoclonal primary antibody (total Nbt1-GFP levels),or using an α-ubiquitin monoclonal primary antibody (α-Ub) (relative ubiquitinated Nbt1-GFP levels). Protein loading was assessed by Panceau S staining of the protein gels. (A) Role of *Cn* Rsp5 in Nbt1 ubiquitination. (B and C) Role of *Cn* arrestin-like proteins in Nbt1 ubiquitination.

The protein with the highest degree of differential ubiquitination in WT cells compared to the *rsp5*Δ and *ali2*Δ mutant strain is a putative purine permease encoded by the gene CNAG_04632 (Fig. 6B). This gene is orthologous to the Dal4 allantoin permease, the Fur4 uracil permease, and the Fui1 uridine permease in *Sc*. We, therefore, refer to this protein as nucleobase transporter 1 (Nbt1) for its likely function in the transport of nucleobase-like compounds. Given the proteomics data suggesting Rsp5- and Ali2-dependent ubiquitination of Nbt1*,* we expressed a GFP-tagged version of this gene in *Cn* WT and in *rsp5*Δ, *ali1*Δ, *ali2*Δ, *ali3*Δ, and *ali4*Δ mutant cells to assess the potential effects of ubiquitination on protein abundance and stability. We also expressed *NBT1-GFP* in an *ali1-4*Δ null background strain lacking all four of the *Cn* arrestin proteins. Additionally, we constructed and expressed in WT cells a GFP-tagged version of the *NBT1* gene with mutation of its ubiquitination site (two lysine residues [K] at positions 546 and 547, mutated to arginine [R]). We assessed the ubiquitination status of this putative nucleobase permease with western blotting, probing with anti-Ub and anti-GFP antibodies. We observed that the Nbt1 protein is only ubiquitinated when Rsp5 is present, and this ubiquitination requires that the two lysine residues comprising the ubiquitination site are intact (Fig. 7A).

The green fluorescent protein tag is moderately resistant toward protease degradation, especially when overexpressed (56). This results in the liberation of the GFP tag when the tagged protein is degraded, which is represented by a ~28 kDa band on a western blot. The western blot data also suggest an association between Nbt1-GFP ubiquitination and protein degradation. The relatively degradation-resistant GFP moiety is liberated in the WT strain, but not so in a mutant lacking Rsp5 nor in the strain expressing Nbt1-GFP with a mutated ubiquitination site (Fig. 7A), indicating that Rsp5-mediated ubiquitination of Nbt1 results in the degradation of this transporter.

We also assessed the degree of Nbt1-GFP ubiquitination and degradation in the arrestin mutant strains. In contrast to the total loss of Nbt1-GFP ubiquitination and degradation in the *rsp5*Δ mutant, we detected residual ubiquitination of the Nbt1 transporter in the *ali1*Δ*, ali2*Δ, *ali3*Δ, *ali4*Δ, and the quadruple *ali1-4*Δ strains, with the latter strain resulting in a more substantial loss of Nbt1 degradation than the single arrestin mutants (Fig. 7B and C). The amount of ubiquitinated Nbt1-GFP protein relative to total Nbt1-GFP protein suggests that Ali2 and other arrestins contribute to but are not required for the ubiquitination and degradation of Nbt1. This result is consistent with a model in which α-arrestins facilitate ubiquitination by directing Ub ligases to their targets.

### Rsp5 and Ali2 directs the localization of Nbt1

**Fig 8:**
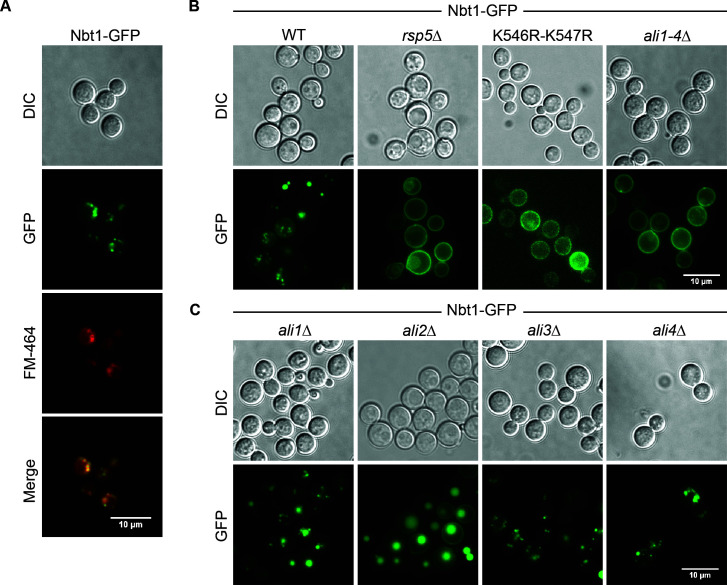
The subcellular localization of GFP-tagged Nbt1. (A) The localization of Nbt1-GFP was assessed with fluorescent microscopy, and localization at perinuclear membranes was assessed by staining cells with the lipophilic dye FM4-64 for 30 minutes. The scale bar indicates 10 µm. (B and C) The localization of Nbt1-GFP was determined with fluorescent microscopy in the indicated strains. The scale bars indicate 10 µm.

We subsequently examined the cellular localization of Nbt1-GFP fusion protein using epifluorescent microscopy. The Nbt1 putative transporter co-localizes in intracellular punctate structures with the FM4-64 fluorescent probe used as a marker for the endocytic pathway, indicating that this protein is likely endocytosed when overexpressed in WT cells (Fig. 8A). However, when expressed in the *rsp5*Δ mutant strain, Nbt1-GFP localizes primarily at the cell surface, suggesting that endocytosis of this protein is disrupted when Rsp5-mediated ubiquitination is perturbed (Fig. 8B). Similarly, cell surface localization is observed in a strain expressing *NBT1-GFP* with a mutated ubiquitination site as well as in the *ali1-4*Δ strain expressing *NBT1-GFP* (Fig. 8C). The putative transporter localizes with a pattern similar to WT in the *ali1*Δ, *ali3*Δ, and *ali4*Δ strains. However, GFP signal predominantly localizes in a large organelle, likely the vacuole, in the *ali2*Δ strain expressing GFP-tagged *NBT1*. These results suggest that Nbt1-GFP is not efficiently endocytosed and subsequently degraded in the absence of Rsp5-directed ubiquitination, or the absence of all four *Cn* α-arrestin-like proteins.

### Nbt1 is one of several putative nucleobase transporters ubiquitinated by Rsp5

Functional grouping of the proteins differentially ubiquitinated in the absence of Rsp5 and Ali2 into GO terms revealed that the transmembrane transporter molecular function term (GO:0022857) was the most represented GO term. Further analysis with the FungiFun2 systematic analysis tool indicated that allantoin and allantoate transport is the most represented term in this group of differentially ubiquitinated proteins (Fig. 6A) and is represented by four genes, including *NBT1*. We therefore refer to the remaining three genes as *NBT2* (CNAG_00749), *NBT3* (CNAG_01690), and *NBT4* (CNAG_04142). While Nbt1 belongs to the nucleobase:cation symporter-1 family of transporters, Nbt2, Nbt3, and Nbt3 belong more broadly to the major facilitator superfamily of transporters, with Nbt3 and Nbt4 sharing an amino acid sequence similarity of 57.5%.

**Fig 9:**
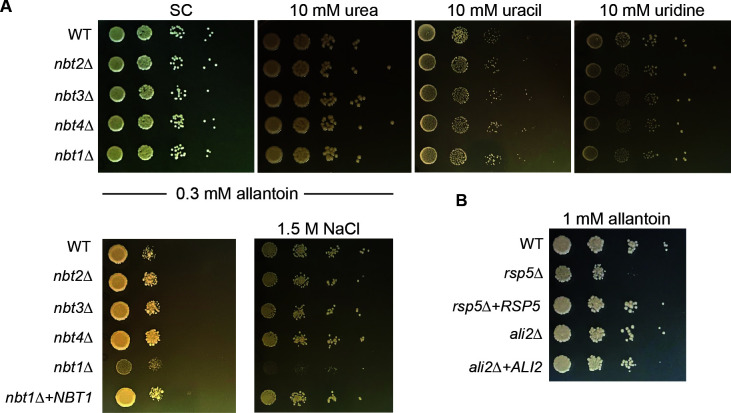
A group of proteins that require both Ali2 and Rsp5 for their ubiquitination and that were predicted to be involved in allantoin transport was tentatively named Nbt1-4. (A) The ability to grow on YNB medium with 10 mM urea, uracil, uridine, and 0.3 mM allantoin (with or without 1.5 M NaCl) as the only nitrogen source was assessed by incubating the indicated strains as serial spot dilutions on these media. A re-constituted strain expressing wild-type *NBT1* (*nbt1*Δ + *NBT1*) was plated on YNB medium with 0.3 mM allantoin. (B) For comparison, the WT, *rsp5*Δ, and *ali2*Δ mutant strains were also incubated as serial spot dilutions on YNB medium with 1 mM allantoin as the only nitrogen source.

To determine if these putative transporter proteins have individual or overlapping roles in purine/nucleobase import, we incubated the strains with mutations in each of the *NBT* genes on synthetic medium with no nitrogen sources except either 10 mM urea, uracil, uridine, or 0.3 mM allantoin, including synthetic complete medium as control. We also incubated these strains on medium with 0.3 mM allantoin as the only nitrogen source with the addition of 1.5 M NaCl to determine if this cellular stress affects the function of these putative transporters. The mutants of these four nucleobase transporters did not display growth defects with urea, uracil, or uridine as the only nitrogen source. However, the *nbt1*Δ mutant has a growth defect compared to WT with allantoin as the only source of nitrogen, irrespective of the presence of NaCl, suggesting that Nbt1 serves a predicted, conserved role in allantoin acquisition (Fig. 9A). Introducing a WT copy of the *NBT1* gene complemented this mutant’s growth defect on medium with allantoin as the only nitrogen source (Fig. 9A). Similar to the *nbt1*Δ mutant, the *rsp5*Δ strain, and not the *ali2*Δ strain, displayed a growth defect with allantoin as the sole nitrogen source (Fig. 9B).

## DISCUSSION

Post-translational modifications of proteins regulate protein activity by altering binding sites, localization, or protein stability. These events are therefore essential components of many intracellular signaling pathways regulating the rapid cellular responses to changing environmental conditions. This is especially true for microbial pathways involved in host interactions and pathogenesis. Therefore, various aspects of post-translational modification have been the focus of new antimicrobial target identification approaches. One such a novel antimicrobial is waldiomycin, an inhibitor of the bacterial WalK histidine kinase that regulates post-translational modifications and viability in Gram-positive bacteria such as *Staphylococcus aureus* and *Bacillus subtilis* (57).

Unlike bacteria, a persisting challenge with all potential targets of antimicrobials for eukaryotic pathogens is that fewer targets exist that do not also have a close homolog in mammalian cells. Here, we report on the fungal E3 Ub ligase, Rsp5, an important post-translational modifier for stress adaptation during host infection. This protein is involved in protein ubiquitination, a form of protein regulation widespread in all eukaryotes and even in some prokaryotes (58). The process of protein ubiquitination involves the transfer of a Ub subunit to a target protein through an E1-E2-E3 enzyme cascade, consisting of activating (E1), conjugating (E2), and ligase (E3) enzymes (59). Specificity of target protein selection is partially directed by this “pyramid” cascade of E1-E2-E3 enzymes. Typically, a single E1 enzyme initially binds to Ub and charges the cysteine residue of a wider range of E2 Ub conjugating enzymes with a Ub unit (60–62). The Ub-charged E2 conjugating enzyme then interacts with one of an even larger pool of E3 ligases, each with binding affinities to different sets of target proteins (63). A Ub subunit is typically transferred to lysine residues within target proteins (monoubiquitination) or to the lysine residues or N-termini of preceding Ub units (polyubiquitination) (63).

E3 Ub ligases not only play a role in target selection, but also in the conformation of these polyubiquitin chains. Specific E3 ligases are also often associated with specific deubiquitinating enzymes that antagonize the inactivation of the E3 ligase through autoubiquitination, thereby limiting substrate ubiquitination and adding another layer of regulation (64, 65). Target selection stringency can be enhanced when an adaptor protein assists E3 ligase and target interaction (66).

Although the ubiquitination cascade is highly conserved, the amino acid sequences and thus exact processes of protein ubiquitination differ between species as well as the multitude of different ubiquitination enzymes that can take part in this cascade. For example, fungal Rsp5 orthologs share definable but limited sequence homology with mammalian E3 Ub ligases in the Nedd4 family. Moreover, a previous study reported that human Nedd4-1 is unable to rescue the phenotypes of a *Sc rsp5*Δ conditional mutant, suggesting functional divergence between the fungal and mammalian enzymes (39). In our hands, preliminary data also suggest that the activity of *Cn* Rsp5 is not inhibited by indole-3-carbinol, a potent inhibitor of human Nedd4-1, since the maximally soluble concentration of this compound does not result in alterations of Rsp5-dependent phenotypes in WT *Cn* (67). Furthermore, unlike the case for *Sc*, Rsp5 is not essential in *Cn*, suggesting some divergence of function between the two fungal Rsp5 proteins but also making this fungus an interesting model to study Rsp5 ubiquitination biology.

Our data suggest that the *Cn* Rsp5 Ub ligase mediates several cell processes through the ubiquitination of a set of target proteins when specific conditions are encountered. We found that *Cn* Rsp5 is required for the ubiquitination of proteins likely involved in nucleobase import. Prior studies found that urea utilization as nitrogen source, as well as *de novo* purine and pyrimidine synthesis, results in the induction of capsule synthesis genes (68, 69). Urea utilization and efficient nucleobase synthesis are also required for *Cn* survival during thermal and other stresses, suggesting that the uptake of urea can serve as a stress response signal. However, the import of ribonucleotides from the environment is not required for survival during stress, and downregulation of these importers might arise from urea-based signaling during stress (70). Studies on nucleotide importers in *Sc*, such as Fur4, suggest that these nitrogen transporters are also ubiquitinated by Rsp5, providing a mechanism to regulate transport activity (71).

Additionally, we demonstrate that Rsp5 is required for the ubiquitination of the chitin synthases Chs4 and Chs5. A previous study on the *S. cerevisiae* Chs3 suggests that this chitin synthase is also ubiquitinated by Rsp5 (72). Altered chitin synthase activity may be the cause of the decreased chitin and chitosan content observed in the *Cn rsp5*Δ mutant, resulting in a defective cell wall. Additionally, some proteins predicted to be involved in cell wall synthesis might be able to bind to Rsp5 directly through their multiple PxY motifs and perhaps without the need for an adaptor protein. A previous study suggested that PxY motifs might be hidden when proteins are in a native conformation but could be exposed when proteins become misfolded during cellular stress (12).

Several *Cryptococcus*-specific phenotypes involved in stress tolerance and pathogenesis are dependent on intact cell wall integrity for their proper expression. For example, the surface polysaccharide capsule requires anchoring by cell wall α-glucans to be maintained on the cell surface (73). Additionally, *Cn* strains with defects in the Rim/alkaline pH response pathway display altered cell wall organization and similarly fail to maintain surface capsule efficiently (74). Moreover, cryptococcal melanin, another important pathogenesis trait, is deposited in the cell wall, and defective cell wall structure often leads to poor melanization (75). Lastly, Szopinska and co-workers showed that several dozen membrane proteins are rapidly endocytosed upon NaCl exposure, likely signaled through their ubiquitination (76). This Ub-mediated change in surface protein localization has been posited as a possible mechanism to rapidly attenuate the effects of ionic/osmotic stress before transcriptional changes can take effect, perhaps anchoring the cell wall to the membrane through membrane protein intermediates. Therefore, the altered cell wall composition/organization demonstrated in the *Cn rsp5*Δ mutant may explain the alterations in capsule and melanin observed in this strain. Modulation of the cell wall through the activity of endochitinases is also required for mating (77). Interestingly, loss of the deubiquitinase Ubp5 results in a 10-fold reduction in mating factor α expression, suggesting a role for ubiquitination in filamentation and mating (78). This finding is in line with our observation that the *rsp5*Δ mutant strain is sterile in an unilateral cross, further suggesting that ubiquitination is an important regulator of processes required for mating in *Cn*.

We have proposed a model in which the *Cn* Rsp5 Ub ligase interacts with its protein targets in a manner facilitated by adaptor proteins. In *Sc*, these adaptors often contain arrestin domains (79). We have previously described four arrestin domain containing proteins in *Cn* (Ali1, Ali2, Ali3, and Ali4) and found that Ali1 interacts with Rsp5, directing this Ub ligase to fatty acid synthases during cell division (28). In this study, we found that Ali2 also interacts with Rsp5, directing it to recognize 38 different protein targets. This assay revealed that Ali3 also likely interacts with Rsp5, since it requires Rsp5 for its ubiquitination.

The high degree of overlap between proteins requiring *Cn* Rsp5 for ubiquitination and those requiring Ali2 for ubiquitination strongly support a model in which the arrestin-like Ali2 protein mediates the interaction of Rsp5 with a specific subset of its targets. This model is also supported by the observation that the *ali2*Δ mutant shares a subset of phenotypes with the *rsp5*Δ mutant when exposed to various cell surface stresses. The patterns of cellular localization for one common target protein for Rsp5 and Ali2, the Nbt1 nucleobase transporter, suggest that Rsp5-mediated ubiquitination is required for the localization and function of Nbt1. Second, the Ali1-4 protein family likely serves overlapping and partially redundant roles in facilitating Nbt1 localization. However, Ali2 appears to play the major role in this adaptor protein family to mediate Nbt1 localization as well as overall pathogenesis.

Rsp5 also mediates the ubiquitination of proteins in an Ali2-independent manner. The majority of proteins with Rsp5-dependent ubiquitination do not, in fact, depend on Ali2 for this post-translational modification. This is likely reflected in the more severe stress-related phenotypes of the *rsp5*Δmutant strain compared to the *ali2*Δ mutant. Ali2-independent targets of Rsp5 include the Cna1 catalytic subunit of the calcineurin phosphatase and two enzymes involved in the trehalose biosynthesis, Tps1 and Tps2 (Table S2). Both calcineurin and intact trehalose homeostasis are required for thermos tolerance, suggesting mechanisms for the poor growth of the *rsp5*Δ mutant strain at high temperature (80, 81).

The severity of the cell wall defects observed in the *Cn rsp5*Δ mutant strain strongly reflects the central role of this Ub ligase in cell wall and cell membrane homeostasis. We found that the survival of *Cn* in the presence of several stressors as well as pathogenesis is dependent on Rsp5-mediated ubiquitination, a novel observation in a pathogenic fungus. This Ub ligase is required for intact cell wall integrity, but the exact cellular mechanism behind this observation remains to be determined. Rsp5 is required for the ubiquitination of several cell wall biosynthesis enzymes, including Fks1, Chs4, Chs5, and Skn1. Therefore, Rsp5 might affect cell wall integrity through modulation of cell wall synthesis protein activity or through localization changes.

## MATERIALS AND METHODS

### Strains, media, and growth conditions

All mutant strains used in this study were generated in the *C. neoformans* var. *grubii* H99 (*MAT*α) (82) or KN99 (*MAT*a and *MAT*α) strain backgrounds (83). Details about these strains are included in Table S4, including mutant strains obtained from Dr. Hiten Madhani (84). All strains were recovered from glycerol stocks stored at −80°C and were routinely maintained on YPD medium (1% yeast extract, 2% peptone, 2% dextrose, and 2% agar for solid medium). Unless otherwise indicated, strains were incubated in YPD medium at 30°C with shaking at 150 rpm. The construction of strains used in this study is described in File S1, with single guide RNA sequences listed in File S2. Oligonucleotide primers used for strain validation and construction are listed in Table S5.

Capsule induction was performed by incubation with shaking in CO_2_-independent medium (CIM; Gibco) at 37°C for 72 h before microscopy (Zeiss Axio Imager A1) observation. Melanin production was assessed on L-DOPA medium (7.6 mM L-asparagine monohydrate, 5.6 mM glucose, 22 mM KH_2_PO_4_, 1 mM MgSO_4_.7H_2_O, 0.5 mM L-DOPA, 0.3 µM thiamine-HCl, 20 nM biotin, pH 5.6) after incubation for 48 h. Survival in the presence of stress was assessed through a spot-dilution assay on supplemented YPD medium as previously described (28). To test for cell surface stress resistance, fungal cells were serially diluted and spotted onto solid media plates and exposed to various cellular stresses, including CR (interferes with cell wall glucan assembly) (85), CFW (interferes with chitin fibril assembly) (86), alkaline pH (disrupts electrochemical gradient) (87), sodium dodecyl sulfate (disrupts cell membranes) (88), NaCl (provides osmotic/ionic stress) (89), caffeine (interferes with the protein kinase C cell wall integrity pathway) (90), and high temperature (wide range of effects, including denaturation of proteins) (91). Nitrogen source utilization was assessed with a spot-dilution assay on media containing single nitrogen sources (yeast nitrogen base medium [YNB] without amino acids/without ammonium sulfate [Gibco] [6.7 g/L], 2% dextrose, 2% Bacto-Agar; plus one of the following nitrogen sources: urea [10 mM], uracil [10 mM], uridine [10 mM], or allantoin [0.3 mM or 1 mM]) after incubation for 48 h. The ability to mate and form filaments were assessed by co-culture of *MAT*α and *MAT*a strains on Murashige and Skoog (MS) medium minus sucrose (Sigma-Aldrich, Steinhelm, Germany).

### Cell wall component quantification, staining, and flow cytometry

The WT strain (H99), the *ali2*Δ mutant strain (KS96-2), and the *rsp5*Δ mutant strain (MDP01) were incubated in liquid YPD medium for 18 h. Cultures were then diluted 10× in fresh YPD medium or fresh YPD medium supplemented with 1.5 M NaCl and conditioned for 2 h (*n* = 2 for each strain). The cells were harvested and fixed in 3.5% formaldehyde at room temperature for 5 minutes and analyzed with flow cytometry and microscopy as previously described (46).

The WT strain (H99), *rsp5*Δ (MDP01), *chs4*Δ (SKE91), and *skn1*Δ (MDP75) mutant strains were incubated in liquid YPD medium for 18 h and then diluted 10× in fresh YPD medium or fresh YPD medium supplemented with 0.5 M NaCl for 48 h (*n =* 3 for each strain). Cells were harvested, flash frozen in dry ice, and lyophilized, and around 50 mg of dry cells were used to quantify chitin and chitin + chitosan using the 3-methyl-benzothiazolinone hydrazine hydrochloride method as previously described (47). Similarly, the β-glucan content in dry cells was quantified using the Megazyme yeast β-glucan kit as previously described (46). The amounts of chitin, chitin + chitosan, and β-glucan were standardized to the amounts of dried cells used. Statistical analysis and plotting of results were performed using GraphPad Prism software (San Diego, CA, USA).

### Survival experiments in mouse and *Galleria mellonella* models

Female and male BALB/c mice were acquired from Charles River Laboratories. Mice (female [*n* = 5], male [*n* = 5]) were anesthetized with 2% isoflurane utilizing a rodent anesthesia device (Eagle Eye Anesthesia, Jacksonville, FL) and were infected via the intranasal route with 10^4^ CFU of either the WT strain (H99), the *ali2*Δ mutant strain (KS96-2), the *ali2*Δ + *ALI2-GFP* strain (CLT67), the arrestin null mutant strain (*ali1*Δ *ali2*Δ *ali3*Δ *ali4*Δ) (CLT57), and the arrestin null mutant strain complemented with the *ALI2* gene (*ali1*Δ *ali2*Δ *ali3*Δ *ali4*Δ + *ALI2-GFP*) (CLT116) in 30 µL of sterile phosphate-buffered saline (PBS). Mice were monitored twice daily for signs of infection and sacrificed at predetermined clinical endpoints that predict mortality for the experimental duration of 50 days. Survival curves were statistically analyzed by log-rank test with Bonferroni correction (GraphPad Prism software).

To assess the role of Rsp5 in virulence, *n =* 10 female C57BL/6 mice (Charles River Laboratories) were infected by inhalation similar to above with 10^4^ CFUs of the WT (H99), *rsp5*Δ mutant (MDP01), or *rsp5*Δ *+ RSP5* (MDP26) complemented strains. The mice were either followed for clinical endpoints predicting imminent mortality (survival experiments) or sacrificed at predetermined times to assess fungal burden by quantitative culture of homogenized whole lungs. Survival curves were statistically analyzed by log-rank test with Bonferroni correction and statistical differences in CFUs recovered from mouse lungs calculated using the Mann-Whitney U-test (GraphPad Prism software).

We infected 30 *Galleria mellonella* larvae (Vanderhorst, St. Marys, OH, USA) for each cryptococcal strain by injecting 10^7^ fungal cells into the protopod of each larva. Larvae were incubated at 30°C and monitored daily for signs of mortality (melanization, lack of movement after prodding). We excluded larvae that transitioned to the pupa state during the infection. Survival data were plotted using a Kaplan-Meier plot, and statistics were performed using the log-rank test (GraphPad Prism software) (92).

### Ali2-GFP proteomic experiment and analysis

This experiment was performed using methods described previously (28). In brief, the WT strain (H99) (*n* = 1) and the *ali2*Δ + *ALI2-GFP* strain (CLT67) (*n* = 3) were incubated in liquid YPD medium for 18 h. Cultures were normalized to an optical density at 600 nm (OD_600_) of 1, resuspended in fresh YPD medium, and incubated for 3 h. To collect total cell lysates, cultures were pelleted, flash frozen on dry ice, and lysed by bead beating. Lysates were collected in 1.4 mL NP40 lysis buffer (6 mM Na_2_HPO_4_, 4 mM NaH_2_PO_4_, 1% Nonidet P-40, 150 mM NaCl, 2 mM EDTA, 1× protease inhibitors [Complete mini, EDTA-free; Roche], 1× phosphatase inhibitors [Phos-Stop; Roche], and 1 mM phenylmethylsulfonyl fluoride), as described previously (44). The crude lysate was cleared by centrifugation at 2,500  ×  *g* at 4°C for 5 minutes, and the supernatant (total cell lysate) was collected. Total cell lysate protein concentrations were measured and normalized using a bicinchoninic acid (BCA) assay. Immunoprecipitations from normalized total cell lysates were performed by the addition of 25 µL GFP-TRAP agarose beads (ChromoTek, Proteintech, Germany) and inversion at 4°C for 2 h.

Mass spectrometry analysis was performed on immunoprecipitations by the Duke Proteomics and Metabolomics Core Facility, as described previously (28). The resin-bound immunoprecipitated proteins were treated with 1% RapiGest (Waters) at 50°C for 10 minutes, reduced by the addition of 10 mM dithiothreitol and incubation at 32°C for 35 minutes, and finally alkylated by the addition of 20 mM final concentration iodoacetamide and incubation at room temperature for 30 minutes. Subsequently, samples were trypsin digested on resin overnight at 32°C. Samples then underwent a 90-minute chromatographic separation using a nanoscale capillary reverse phase ultra-performance liquid chromatography system (Waters) in combination with a Q-Exactive Plus high-resolution accurate mass tandem mass spectrometer (Thermo Scientific) via a nano-electrospray ionization source.

Proteins identified through this method were prioritized by strength and specificity of their potential interactions with Ali2-GFP, as previously described (28). In brief, the average exclusive unique peptide count was averaged across the three *ali2*Δ + *ALI2-GFP* immunoprecipitations for each identified protein. The percent of the APC identified in the WT immunoprecipitation was then calculated for each identified protein. Identified proteins with an APC ≥2 and an APC percent identified in the WT immunoprecipitation <20% were designated as prioritized potential interactors of Ali2-GFP.

### Differential ubiquitination experiment and analysis

The WT strain (H99) (*n* = 3), the *rsp5*Δ mutant strain (MDP01) (*n* = 3), and the *ali2*Δ mutant strain (KS96-2) (*n* = 3) were incubated in liquid YPD medium for 18 h. Cultures were normalized to an OD_600_ of 3 in fresh YPD medium and fresh YPD medium supplemented with 1.5 M NaCl and conditioned for 1 h. Samples were washed twice with PBS and flash frozen on dry ice.

Sample preparation and mass spectrometry analysis were performed by the Duke Proteomics Core Facility. In short, cell pellets were treated with 8 M urea and subsequently lysed by bead beating. Total cell lysates were collected, protein content was measured by Bradford Assay, and normalized proteins were digested to peptides. Ubiquitinated peptides were enriched using the PTMScan HS Ubiquitin/SUMO Remnant Motif (K-ε-GG) Kit (Cell Signaling Technology). Quantitative LC/MS/MS was performed on isolated peptides using a nanoAcquity UPLC system (Waters) coupled to a Thermo Orbitrap Fusion Lumos high-resolution accurate mass tandem mass spectrometer (Thermo Scientific) via a nano-electrospray ionization source. Ubiquitinated peptide expression values were used to calculate fold changes between experimental samples. Data were statistically analyzed by two-tailed *t*-tests on log_2_ transformed values. All peptides and their corresponding proteins that were determined to have a twofold reduction in ubiquitination in either mutant strain background were used for further analysis by FunCat enrichment analysis using default settings, including a 5% false discovery rate with a Benjamini-Hochberg correction (51). Differentially ubiquitinated peptides were plotted using the ggplot package in the R coding language and protein sequences were screened for PxY motifs in the R coding language using RStudio.

### Protein purification and immunoblotting

The WT strain (H99), the *ali2*Δ mutant strain (KS96-2), and the *rsp5*Δ mutant strain (MDP01) were incubated in liquid YPD medium for 18 h. Cultures were washed twice in PBS and subsequently normalized by OD_600_. Cells were conditioned in fresh YPD medium, fresh YPD medium supplemented with 1 M NaCl, or fresh YPD medium supplemented with 1 mg/mL caffeine for 3 h. Total cell lysates were prepared as previously described (93). Total cell lysate protein concentrations were measured and normalized using a BCA protein quantification assay (Thermo Scientific).

For western blotting analysis, samples were diluted in 4× lithium dodecyl sulfate (LDS) loading sample buffer and boiled for 5 minutes, and western blots were performed as previously described (93). To detect phosphorylated Hog1, blots were incubated with an anti-phosphorylated p38 Hog1 polyclonal primary antibody (1/1,000 dilution; Cell Signaling Technology, 9211S; lot 25) and an anti-rabbit peroxidase-conjugated secondary antibody (1/50,000 dilution; Jackson ImmunoResearch Laboratories, Inc., 115-035-174; lot 127837). To detect histone H3 as a loading control, blots were incubated with an anti-H3 (D1H2 polyclonal, Cell Signaling Technology) and anti-rabbit-HRP monoclonal primary antibody (1/1,000 dilution; Sigma, P7962; lot 015M4840V). To detect phosphorylated Mpk1, blots were incubated with a phospho-p44/42-MAPK antibody (1/1,000 dilution, Cell Signaling Technology, Beverly, MA, USA, #4511) and an anti-rabbit peroxidase-conjugated secondary antibody as before. The blot was stripped with incubation in Restore western blot stripping buffer (Thermo Scientific) for 15 minutes, followed by blocking and washing, as described before (93). Proteins were detected by enhanced chemiluminescence (ECL Prime Western blotting detection reagent; GE Healthcare).

The WT strain (H99) and all strains expressing GFP-tagged *NBT1* were incubated in liquid YPD medium for 18 h. Cultures were washed twice in PBS and subsequently normalized by OD_600_. Total cell lysates were prepared as before and normalized cell lysates were immunoprecipitated by adding to 25 µL GFP-TRAP agarose bead suspension (ChromoTek) equilibrated in NP-40 lysis buffer, as described previously (94). Protein eluded from the GFP-TRAP beads was added to LDS loading buffer and incubated at room temperature for 30 minutes. Western blotting was performed as earlier. To detect ubiquitinated protein, blots were incubated with an anti-ubiquitin monoclonal-horse radish peroxidase-conjugated antibody (1/1,000 dilution; Cytoskeleton, Inc., #AUB01; lot 033). To detect *NBT1-*GFP, the blots were stripped with incubation in Restore western blot stripping buffer (Thermo Scientific) for 15 minutes, washed and blocked as before, and re-probed with an anti-GFP monoclonal primary antibody (1/5,000 dilution; Roche Applied Science, 11814460001; lot 14717400). An anti-mouse peroxidase-conjugated secondary antibody (1/25,000 dilution; Jackson ImmunoResearch Laboratories, Inc., 111-035-008; lot 128022) was used to allow detection by enhanced chemiluminescence.

### Microscopy

All strains expressing GFP-tagged *NBT1* were incubated in liquid YPD medium for 18 h. Cells were harvested and washed with PBS and resuspended in PBS. Cells from strain MDP49 (*NBT1*-GFP-NAT) were stained with the lipophilic dye, FM4-64 (Molecular Probes), at 1/1,000 dilution in PBS, Molecular Probes, for 30 minutes at room temperature. The MDP49 cells were washed with PBS and resuspended in PBS. Differential interference microscopy and fluorescent images were visualized with a Zeiss Axio Imager A1 fluorescence microscope (60× or 100× objectives) using a GFP filter for GFP fluorescence. Images were taken with an AxioCam MRm digital camera with ZEN Pro software (Zeiss). FM4-64 staining was visualized using a Texas Red filter. The same exposure time was standardized across all strains and images were analyzed using ImageJ/Fiji software (95).

## Data Availability

The differential ubiquitination data analyzed in this publication have been deposited at the Eukaryotic Pathogen, Vector & Host Informatics Resources (https://VEuPathDB.org) Bioinformatics Resource Center. All other data are available on request.

## SUPPLEMENTAL MATERIAL

File S1 mbio.00981-24-s0001.pdf Strain construction methods.

File S2 mbio.00981-24-s0002.pdf Single guide RNA sequences used for CRISPR-mediated strain construction.

Table S1 mbio.00981-24-s0003.xlsx Protein interactors of Ali2-GFP.

Table S2 mbio.00981-24-s0004.xlsx Proteins that require Rsp5 for their ubiquitination.

Table S3 mbio.00981-24-s0005.xlsx Proteins that require Ali2 for their ubiquitination.

Table S4 mbio.00981-24-s0006.xlsx Strains used.

Table S5 mbio.00981-24-s0007.xlsx Oligonucleotide primers used.

ASM does not own the copyrights to Supplemental Material that may be linked to, or accessed through, an article. The authors have granted ASM a non-exclusive, world-wide license to publish the Supplemental Material files. Please contact the corresponding author directly for reuse.
