# Supplementary material for: A fungal ubiquitin ligase and arrestin binding partner contribute to pathogenesis and survival during cellular stress
Source: mBio. 2024 Sep 5;15(10):e00981-24. doi: 10.1128/mbio.00981-24 (PMC11481503; doi:10.1128/mbio.00981-24)
Supplement: File S1 — Strain construction methods. [file mbio.00981-24-s0001.pdf]

Supplemental File 1.

Strain Construction Method

The *ali2*Δ mutant strain (KS96-2) was complemented by PCR amplifying the *ALI2* gene from WT *C. neoformans* H99 genomic DNA in addition to 500 base pairs (bp) upstream and downstream from the open reading frame (ORF) using primer pair AA5405 and AA5406. The pJAF1 plasmid (Fraser et al., 2003) with a neomycin resistance gene (NEO) was PCR amplified using primer pair AA5403 and AA5404 and the *ALI2* gene with 500 bp upstream and downstream regions was cloned into the pJAF1 plasmid, giving plasmid pCT6. The resulting plasmid was transformed into *ali2*Δ mutant cells with biolistic transformation, as previously described (Toffaletti et al., 1993). Transformants were screened for neomycin resistance and for the presence of the WT *ALI2* allele with PCR amplification.

The *rsp5*Δ mutant strain (MDP01) was generated by CRISPR/Cas9-based gene targeting via gene replacement with a split dominant nourseothricin resistance gene (NAT) through homologous recombination (HR) (Kim et al., 2012). A split NAT resistance gene fused to fragments homologous to 1 kb upstream and downstream of the WT (H99) *RSP5* gene was used as the HR construct. The 5'-end of the split marker was PCR amplified with the primer pair AA5557 and AA4096 from the pCH233 plasmid (McDade & Cox, 2001), containing the NAT resistance gene under the control of the WT (H99) actin (*ACT1*) promoter and the phosphoribosyl anthranilate isomerase (*TRP1*) terminator. Primer pair AA5552 and AA5556 was used to PCR amplify 1 kb upstream of the *RSP5* gene from WT (H99) genomic DNA and fused to the 5'-end of the NAT resistance gene with primer pair AA5552 and AA4096 using cloning-free stitching PCR. The 3'-end of the NAT resistance gene was PCR amplified with primer pair AA4097 and AA5558 from plasmid pCH233. Primer pair AA5559 and AA5560 was used to PCR amplify 1 kb downstream of the *RSP5* gene from WT (H99) genomic DNA and fused to the 3'-end of the NAT resistance gene with primer pair AA4097 and AA5560.

The *Streptococcus pyogenes CAS9* gene under the control of the WT (H99) glycerol-3-phosphate dehydrogenase (*GPD1*) promoter and terminator was PCR amplified from the

pXL1-Cas9-HYG plasmid (Fan & Lin, 2018) with primer pair AA5257 and AA5258. The single guide RNA (sgRNA) DNA fragment under the control of the *C. deneoformans* JEC21 U6 promoter and 6-T terminator was designed using the Eukaryotic Pathogen CRISPR guide RNA/DNA Design Tool (Peng & Tarleton, 2015) and bought as a gBlock oligonucleotide fragment from Integrative DNA Technologies (IDT) (see **Supplemental file 2** for the DNA sequence). The sgRNA DNA fragment was PCR amplified with the primer pair AA5503 and AA5506. The CRISPR/Cas9 components were introduced into WT (H99) cells via electroporation, as described previously (Fan & Lin, 2018).

The *rsp5*Δ + *RSP5* strain (MDP26) was made by reintroducing the WT (H99) *RSP5* gene into the native locus in *rsp5*Δ mutant cells (MDP01). The CRISPR/Cas9-based approach described earlier was used with a sgRNA DNA fragment targeting the NAT resistance marker. The HR construct was made by fusing two fragments consisting of the intact *RSP5* gene and 1 kb upstream and downstream of the gene. A single base substitution introduced by one primer allowed for distinguishing between successful transformants and WT (H99) contamination. The 5'-fragment was PCR amplified from WT (H99) genomic DNA with primer pair AA5552 and AA5570 and the 3'-fragment was amplified with primer pair AA5571 and AA5560. The fragments were fused with cloning-free stitching PCR with primer pair AA5552 and AA5560. The remaining CRISPR/Cas9 components were constructed as described previously and introduced into the *rsp5*Δ mutant strain (MDP01) with electroporation. Transformants were screened for the ability to grow at 39°C, as *rsp5*Δ mutant cells are unable to grow at this temperature. Selected colonies were confirmed with PCR amplification of the *RSP5* ORF and Sanger sequencing.

The *rsp5*Δ mutant strain (*MAT***a**) (MDP11) was generated by genetic crossing between *rsp5*Δ (*MAT*α, MDP01) and KN99a on Murashige and Skoog (MS) medium minus sucrose (Sigma-Aldrich, Steinhelm, Germany) (Murashige & Skoog, 1962). Progeny were screened for NAT resistance and subsequently confirmed by PCR amplification.

The *ali1*Δ *ali2*Δ *ali3*Δ *ali4*Δ + *ALI2*-GFP strain (CLT116) was constructed by genetic crossing between *ali1*Δ *ali2*Δ *ali3*Δ *ali4*Δ (CLT57) and *ali2*Δ + *ALI2*-GFP (CLT67) on MS

medium. The progeny was screened by NAT resistance, NEO resistance, and epifluorescence microscopy and subsequently confirmed by PCR amplification and Sanger sequencing.

Primer pair AA5677 and AA5680 was used to validate that the *NBT1* ORF was removed from mutant *nbt1*Δ (strain MDP58). Similarly, primer pair AA5685 and AA5686 was used to validate *NBT2* ORF removal (MDP59), pair AA5683 and AA5684 for *NBT3* (MDP60) and pair AA5706 and AA5707 for *NBT4* (MDP61). Primer pair AA5669 and AA5670 was designed with regions overlapping plasmid pGWKS10 (Spencer et al., 2020) and primer pair AA5667 and AA5668 was designed with regions overlapping the *NBT1* gene. These primers were used to PCR amplify the NBT1 gene and plasmid pGWKS10, respectively and the resulting fragments were assembled using the NEBuilder DNA assembly kit, creating plasmid pMDP05. Similarly, primer pair AA5697 and AA5698 was used to mutate lysine residues at positions 546 and 547 to arginine in plasmid pMDP05, creating plasmid pMDP06 through assembly with the NEbuilder DNA assembly kit. These plasmids were introduced into WT (H99) cells with biolistic transformation as described earlier, creating strains MDP49 with *NBT1* fused to the clover (GFP) fluorescent marker and MDP54 with *NBT1* fused to the clover (GFP) fluorescent marker and K546 and K547 mutated to arginine. The *NBT1*-Clover (GFP) fusion was PCR amplified and verified with Sanger sequencing.

The *nbt1*Δ + *NBT1* strain (MDP105) was made similarly to the *rsp5*Δ + *RSP5* strain (MDP26), using a CRISPR/Cas9-based approach to reintroduce a wild type copy of the *NBT1* gene into the native locus. Primer pair AA5718 and AA5723 was used to amplify the *NBT1* gene from *Cn* H99 genomic DNA, including 1 kb upstream and 1 kb downstream of the gene. The CRISPR/Cas9 components were constructed as described previously and introduced into the *nbt1*Δ mutant strain (MDP58) with electroporation. As no selectable marker was used alongside the *NBT1* gene, transformants were screened for the ability to grow on SC agar plates with allantoin as the only nitrogen source, as *nbt1*Δ mutant cells are unable to grow with allantoin as the only nitrogen source. Reconstitution of the gene was validated with PCR and Sanger sequencing.

To create strains MDP53, MDP67, MDP68, MDP69, and MDP70, strains MDP49 (*MAT*α *NBT1*-GFP-NAT) and CLT57 (*MAT***a** *ali1*Δ::NEO *ali2*Δ::NAT *ali3*Δ::NAT *ali4*Δ::NAT) were genetically crossed on MS medium and resulting progeny were screened with PCR amplification. To create strain MDP52, strains MDP49 (*MAT*α *NBT1*-GFP-NAT) and MDP11 (*MAT***a** *rsp5*Δ::NAT) were genetically crossed on MS medium and resulting progeny were screened with PCR amplification.

References

Murashige, T., & Skoog, F. (1962). A revised medium for rapid growth and bioassays with tobacco tissue cultures. *Physiol. Plant*, *15*, 473-497.

Fan, Y., & Lin, X. (2018). Multiple applications of a transient CRISPR-Cas9 coupled with electroporation (TRACE) system in the Cryptococcus neoformans species complex. *Genetics*, *208*(4), 1357-1372.

Fraser, J. A., Subaran, R. L., Nichols, C. B., & Heitman, J. (2003). Recapitulation of the sexual cycle of the primary fungal pathogen Cryptococcus neoformans var. gattii: implications for an outbreak on Vancouver Island, Canada. *Eukaryotic cell*, *2*(5), 1036-1045.

Kim, M. S., Kim, S.-Y., Jung, K.-W., & Bahn, Y.-S. (2012). Targeted gene disruption in Cryptococcus neoformans using double-joint PCR with split dominant selectable markers. *Host-fungus interactions: Methods and protocols*, 67-84.

McDade, H., & Cox, G. (2001). A new dominant selectable marker for use in Cryptococcus neoformans. *Sabouraudia*, *39*(1), 151-154.

Peng, D., & Tarleton, R. (2015). EuPaGDT: a web tool tailored to design CRISPR guide RNAs for eukaryotic pathogens. *Microbial genomics*, *1*(4), e000033.

Spencer, G. W., Chua, S. M., Erpf, P. E., Wizrah, M. S., Dyba, T. G., Condon, N. D., & Fraser, J. A. (2020). Broadening the spectrum of fluorescent protein tools for use in the encapsulated human fungal pathogen Cryptococcus neoformans. *Fungal genetics and biology*, *138*, 103365.

Toffaletti, D. L., Rude, T. H., Johnston, S. A., Durack, D., & Perfect, J. (1993). Gene transfer in Cryptococcus neoformans by use of biolistic delivery of DNA. *Journal of bacteriology*, *175*(5), 1405-1411.
