# Supplementary material for: A fungal ubiquitin ligase and arrestin binding partner contribute to pathogenesis and survival during cellular stress
Source: mBio. 2024 Sep 5;15(10):e00981-24. doi: 10.1128/mbio.00981-24 (PMC11481503; doi:10.1128/mbio.00981-24)
Supplement: File S2 — Single guide RNA sequences used for CRISPR-mediated strain construction. [file mbio.00981-24-s0002.pdf]

Supplemental File 2. Single guide RNA sequences used for CRISPR-mediated strain construction

Single guide RNA targeting *RSP5*

```
Tttgcattagaactaaaaacaaagcatgattattacagttcatttatttttaaattgatcggcatgc
atgcaaagtatacgtgcaaggacaatggtaacctgcaggtgtgaccgataattataaccatttgttga
gaatgaagaggtgaggagaaaaacaatggatgacgggaaaaaaataaaaaaacactgagacggcgtgg
accgccgtcttatttgcttccgttatccgccaaagtggaaattgcacatacaccggcagggtatactg
ttACTGCTTGGGTTGCGGGTTGgttttagagctagaaatagcaagttaaaataaggctagtccgttat
caacttgaaaaagtggcaccgagtcggtgctttttttgttttat
```

Single guide RNA targeting NAT

```
tttgcattagaactaaaaacaaagcatgattattacagttcatttatttttaaattgatcggcatgc
atgcaaagtatacgtgcaaggacaatggtaacctgcaggtgtgaccgataattataaccatttgttga
gaatgaagaggtgaggagaaaaacaatggatgacgggaaaaaaataaaaaaacactgagacggcgtgg
accgccgtcttatttgcttccgttatccgccaaagtggaaattgcacatacaccggcagggtatactg
ttGATCGCCGGTGCGTTGACGTgttttagagctagaaatagcaagttaaaataaggctagtccgttat
caacttgaaaaagtggcaccgagtcggtgctttttttgttttat
```
